# Microbially-accelerated consolidation of oil sands tailings. Pathway I: changes in porewater chemistry

**DOI:** 10.3389/fmicb.2014.00106

**Published:** 2014-03-21

**Authors:** Tariq Siddique, Petr Kuznetsov, Alsu Kuznetsova, Nicholas Arkell, Rozlyn Young, Carmen Li, Selma Guigard, Eleisha Underwood, Julia M. Foght

**Affiliations:** ^1^Department of Renewable Resources, University of AlbertaEdmonton, AB, Canada; ^2^Department of Biological Sciences, University of AlbertaEdmonton, AB, Canada; ^3^Department of Civil and Environmental Engineering, University of AlbertaEdmonton, AB, Canada

**Keywords:** methanogenesis, porewater chemistry, biogeochemical pathways, consolidation, oil sands tailings

## Abstract

Dispersed clay particles in mine tailings and soft sediments remain suspended for decades, hindering consolidation and challenging effective management of these aqueous slurries. Current geotechnical engineering models of self-weight consolidation of tailings do not consider microbial contribution to sediment behavior, however, here we show that microorganisms indigenous to oil sands tailings change the porewater chemistry and accelerate consolidation of oil sands tailings. A companion paper describes the role of microbes in alteration of clay chemistry in tailings. Microbial metabolism in mature fine tailings (MFT) amended with an organic substrate (hydrolyzed canola meal) produced methane (CH_4_) and carbon dioxide (CO_2_). Dissolution of biogenic CO_2_ lowered the pH of amended MFT to pH 6.4 vs. unamended MFT (pH 7.7). About 12% more porewater was recovered from amended than unamended MFT during 2 months of active microbial metabolism, concomitant with consolidation of tailings. The lower pH in amended MFT dissolved carbonate minerals, thereby releasing divalent cations including calcium (Ca^2+^) and magnesium (Mg^2+^) and increasing bicarbonate (HCO^−^_3_) in porewater. The higher concentrations increased the ionic strength of the porewater, in turn reducing the thickness of the diffuse double layer (DDL) of clay particles by reducing the surface charge potential (repulsive forces) of the clay particles. The combination of these processes accelerated consolidation of oil sands tailings. In addition, ebullition of biogenic gases created transient physical channels for release of porewater. In contrast, saturating the MFT with non-biogenic CO_2_ had little effect on consolidation. These results have significant implications for management and reclamation of oil sands tailings ponds and broad importance in anaerobic environments such as contaminated harbors and estuaries containing soft sediments rich in clays and organics.

## Introduction

The oil sands in Alberta, Canada are the world's third largest proven reserve comprising ~170 billion barrels of recoverable bitumen, with production expected to surpass 3.5 million barrels day^−1^ by 2020. Bitumen is extracted from surface-mined ores using hot water and hydrocarbon solvent (Schramm et al., 2000), generating ~1 million m^3^ fluid fine tailings day^−1^ that are deposited and retained in tailings ponds. The current total volume (>920 million m^3^) and surface area (~182 km^2^) of the ponds will continue to increase with resource exploitation (Alberta Environment and Sustainable Resource Development, 2013) (http://www.aer.ca/rules-and-regulations/directives/tailings-plans-2012). Two major challenges plague the oil sands surface mining industry: (1) recovering water from tailings for re-use (7.5–10 barrels of water for every barrel of bitumen produced), so as to reduce the need for fresh water, and (2) consolidating the tailings solids to decrease the volume of stored tailings for subsequent reclamation.

The tailings deposited into ponds (initially at ~10 wt% solids) settle by gravity in 2–4 years to form mature fine tailings (MFT, ~30 wt% solids), a colloidal suspension comprising slightly alkaline water, silt, clay, unrecovered bitumen (~5 wt%) and residual solvent (<1 wt%) such as naphtha, a mixture of C_3_–C_14_ aliphatic and monoaromatic hydrocarbons. MFT then requires decades to consolidate to >60 wt% solids (Fedorak et al., 2003; Jeeravipoolvarn et al., 2009) through close packing of clay particles accompanied by interstitial water (porewater) expression to form a “cap water” layer. Tailings ponds harbor indigenous microbial communities (Penner and Foght, 2010; Siddique et al., 2011, 2012) that anaerobically biodegrade organic compound, such as solvent hydrocarbons, to methane (CH_4_) plus carbon dioxide (CO_2_) (Siddique et al., 2006, 2007, 2011). Biogenic gas production has been associated with accelerated consolidation of MFT and recovery of porewater both *in situ* and in the laboratory (Fedorak et al., 2003; Bressler et al., 2010). To date, only simple physical mechanisms such as creation of transient channels for water transport by biogenic gas bubbles (Brown et al., 2013; Voordouw, 2013) have been proposed to explain this phenomenon, but this mechanism inadequately describes consolidation when gas is formed but ebullition is minimal.

**Figure 1:**
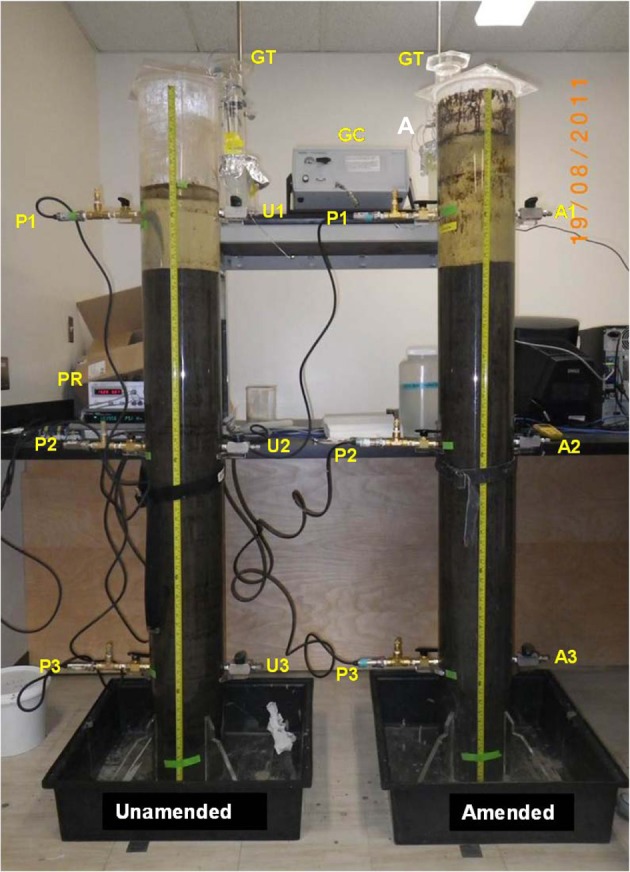
**Experimental 50-L columns used for measuring consolidation, porewater recovery and gas release from oil sands mature fine tailings (MFT) either amended (A) with an organic carbon source or unamended (U)**. The photograph was taken after 105 d incubation at ~20°C. Three sampling ports for collecting MFT or cap water (A1–A3; U1–U3) and three instrumentation ports (P1–P3) fitted with pressure transducers and a pressure reading unit (PR) for recording *in situ* pore pressure were installed in each column; see Methods for details. The columns were sealed under a N_2_ gas atmosphere. Stainless steel tubing connected the headspace to a micro-gas chromatograph (GC) to determine the composition of released biogenic gas, and to gas traps (GT) for measuring cumulative emitted gas volumes.

Because biodensification is slow *in situ* when supported solely by endogenous substrates, we amended MFT with low concentrations of labile organic substrates to enhance anaerobic microbial activity and accelerate the processes *ex situ*. Here we describe a laboratory study conducted using 50-L columns (Figure 1). The results reveal biogeochemical reactions that impact the chemistry of three tailings components—porewater, expressed porewater (cap water) and solids—and accelerate consolidation of MFT. We describe how microbial metabolism alters the chemistry of porewater that in turn influences consolidation of clay particle suspensions. A companion paper (Siddique et al., 2014) describes microbe:mineral interactions in the solids fraction. The combined results and biogeochemical models are relevant for proposed reclamation strategies for oil sands tailings in uplands and end pit lakes.

In addition, the fundamental mechanisms reported here have broad implications for developing geotechnical models to predict behavior of organic-rich saturated soft sediments (i.e., contaminated harbors, and marine, lacustrine and estuarine sediments), predicting contaminant transport from sediments to overlying water, and assessing remedial strategies for containing contaminants through *in situ* sediment capping (Himmelheber et al., 2011).

## Materials and methods

### Preparation of 50-L columns

Mature fine tailings (MFT; Table 1) and cap water were collected from Mildred Lake Settling Basin (MLSB; 57°4′27″N, 111°38′19″W UTM) at Syncrude Canada Ltd. in Fort McMurray, Alberta, Canada in April 2011, and stored at room temperature (~20°C, *ca*. tailings pond *in situ* temperature) for a few days before using for the experiment. The solids and bitumen contents of the MFT were determined using the Dean and Stark method (Dean and Stark, 1920) to be ~35 and ~2.5%, respectively. The solids content was adjusted to 25 wt% solids using tailings pond “cap water” immediately before beginning the experiment, to allow this study to be compared to previous studies (Young et al., in preparation). For each of two parallel 50-L columns (described below), 30 L MFT was combined with 15.45 L tailings pond cap water by gentle but thorough manual mixing under a curtain of N_2_ gas to achieve a solids content of 25 wt% while maintaining anaerobic conditions. For the test column, the added cap water contained soluble canola meal hydrolysate (preparation and composition described in Table 2) sufficient to provide a final concentration of 400 mg C L^−1^ MFT. The cap water used for adjusting MFT in the other column was unaltered, serving as a baseline control in which only endogenous carbon was available to support microbial activity.

**Table 1:** **Chemical characteristics of mature fine tailings used in 50-L column experiment prior to amendment and incubation**.

**Analysis (units); number of replicates**	**Value**
**DEAN-STARK (wt%)^a^; *n* = 1**	
Bitumen	1.5
Water	74.9
Solids	23.4
Total	99.8
**TEXTURE (wt%)^b^; *n* = 1**	
Clay <2 μm	25.6
Silt 2–50 μm	70.9
Fine sand 50–250 μm	3.5
Bulk pH; *n* = 2	7.3 ± 0.03
E_*h*_ (mV); *n* = 3	−2.0 ± 16
**SOLUBLE CATIONS (mg L^−1^ POREWATER)**	
Ca^2+^; *n* = 2	18.1 ± 1.1
Mg^2+^; *n* = 3	8.6 ± 1.2
K^+^; *n* = 2	24.6 ± 2.7
Na^+^; *n* = 2	737.8 ± 37.6
**SOLUBLE ANIONS (mg L^−1^ POREWATER)**	
SO^2−^_4_; *n* = 2	20.6 ± 1.2
Cl^−^; *n* = 3	606.0 ± 21.0
HCO^2−^_3_; *n* = 3	1620 ± 58.5
PO^2−^_4_; *n* = 3	BDL^c^
Carbonates (wt%)^b^; *n* = 2	2.4 ± 0.1

*Mean values of replicates are shown ± 1 standard deviation*.

a *(Dean and Stark, 1920)*.

b *Calculated on oven dry weight basis*.

c *BDL, below detection limit (0.01 mg L^−1^)*.

**Table 2:** **Elemental composition of hydrolyzed canola meal^a^ used as the labile organic amendment in the experiment**.

**Analysis (units); number of replicates**	**Value**
**ELEMENTAL ANALYSIS (mg g^−1^)^b^; *n* = 1**	
Carbon	28.1
Nitrogen	4.3
Hydrogen	4.0
Sulfur	0.4
**CATIONS (mg L^−1^)^c^**	
Ca^2+^; *n* = 4	1.6 ± 0.1
Mg^2+^; *n* = 4	3.1 ± 0.1
K^+^; *n* = 4	15.7 ± 0.2
Na^+^; *n* = 4	291.3 ± 5.6
**ANIONS (mg L^−1^)^c^**	
SO^2−^_4_; *n* = 2	2.3 ± 0.1
Cl^−^; *n* = 2	366.9 ± 11.2
HCO^2−^_3_; *n* = 2	213.3 ± 8.9
PO^2−^_4_; *n* = 2	0.9 ± 0.04

*Mean values of replicates are shown ± 1 standard deviation*.

a *Canola hydrolysate was prepared from dry canola meal (post-pressing to remove oil; provided by Sanimax, Canada) by homogenizing the meal in a mortar and adding 10 g of powder to 90 ml of 4% sodium hydroxide. The slurry was preheated to 60°C for 5 min and then stirred at 180 rpm for 24 h at 60°C. Following hydrolysis, the residue was collected by centrifugation at 9000 g and the supernatant was collected and adjusted to pH 9 with drops of 1 M HCl. The resulting liquid was freeze-dried and stored as a powder at room temperature*.

b *Calculated on oven dry weight basis*.

c *To assess the contribution of the canola meal amendment to soluble cations and anions in MFT porewater and cap water, the hydrolysate was dissolved in nanopure water at the same ratio as that used in the amended MFT (400 mg C L^−1^)*.

Two cylindrical acrylic columns (30.48 cm outer diameter, 0.64 cm wall thickness, and 195.6 cm height; Johnston Industrial Plastics Ltd., Edmonton, AB, Canada) were sealed at the base with acrylic plates and lateral supports (Figure 1). The cylinders were equipped with brass sampling ports (U1–U3 for unamended and A1–A3 for amended columns) for removal of MFT or water samples from the inner portion of the column, and with PX409-005G5V (OMEGADYNE Inc.) pressure transducers (P1–P3) and an Agilent 34972A Data Acquisition/Data Logger pressure reading unit (PR) for measuring pore pressure *in situ*.

The experiment was established on May 6, 2011 by filling each column with 45 L prepared MFT under a plastic curtain flushed with a continuous stream of nitrogen (N_2_) to maintain anaerobic conditions and minimize exposure of MFT to air. In both columns, the adjusted MFT formed a single layer of suspended clays with no free water at the surface. After filling, the columns were sealed under an N_2_ headspace using acrylic plates fitted with gas-tight stainless steel gas ports and tubing (0.15 cm diam.) connecting the headspace to a gas trap and a micro-gas chromatograph to determine the volume and composition of emitted gas, respectively. The columns were incubated in dim natural light at ~20°C. Real time measurements of biogenic gas production and composition, *in situ* pH, porewater recovery and consolidation of tailings were recorded during 213 d incubation. Consolidation and porewater recovery were measured as the height of the cap water-solid interface (the “mud line”) and corrected for trapped gas volume as described below.

At the end of the experiment, cap water and MFT samples were collected from the center of the column via sampling ports, using 4 mm stainless steel tubing and syringes. Samples were analyzed for carbonate minerals, soluble and exchangeable cations, and soluble anions, as described below. These samples were also subjected to solid phase chemical and molecular microbiological analyses described and discussed in the companion manuscript (Siddique et al., 2014).

We were unable to include sterilized MFT as a control condition for several reasons. First, we have observed previously (unpublished results) that it is very difficult to ensure sterilization of the thick MFT suspension, even with lengthy, repeated autoclaving over three or four successive days. Second, we did not wish to alter the structure or chemistry of the tailings by subjecting them to heat, as this could affect consolidation. Finally, we did not have access to a gamma source of sufficient capacity for irradiative sterilization of ~90 L of MFT.

### Small-volume complementary experiments

To observe the effect of non-biogenic (exogenous) gas amendment on consolidation of MFT, four parallel 2-L columns were prepared. Each column was filled with 1.7 L MFT (adjusted to 25% solids) under anaerobic conditions. One column received MFT that had been sparged with N_2_ gas (5.0 Ultra high purity, Praxair) at a flow rate of 62.5 mL min^−1^ for 1 h prior to dispensing into the column. MFT for two replicate columns was sparged with CO_2_ (4.0 Anaerobic, Praxair) at a flow rate of 72 mL min^−1^ for 1 h until pH stabilized between 6.54 and 6.68, then was placed in the columns. MFT for the fourth column did not receive any gas sparging (untreated control). All columns were sealed under an anaerobic (N_2_) headspace and incubated undisturbed at ~20°C in the dark for 120 d. The columns were monitored regularly for expressed porewater recovery (cap water) and solids consolidation by measuring the height of the mud line and water surface and converting to volume.

To test the effect of different cations, ionic strength and pH on the settling behavior of clays particles separately from microbial effects, a small-volume, short-term experiment was conducted in triplicate in 15-mL conical Falcon tubes (Fisher Scientific, Canada). Seven milliliters of MFT was placed in each Falcon tube and amended with 7 mL of one of the following solutions: 1N NaCl; 1N CaCl_2_; 1N FeCl_2_; 0.2M ammonium oxalate buffer (pH 3); 6M HCl (pH 1); or Nanopure water (Barnstead; Thermo Scientific). The tubes were shaken on a reciprocating shaker for 2 h and then placed upright on the bench to consolidate without disturbance. The mud line height in each tube was recorded after 30 min and again after 24 h.

### Analytical methods and calculations

We define here two water compartments analyzed in our experiments: (1) “porewater” is interstitial water associated with the colloidal MFT suspension, and is a component of the MFT; (2) “cap water” is porewater that has been expressed from the MFT and has collected above the mud line during consolidation. The term “mud line” used here is defined as the interface between cap water and MFT.

#### Porewater recovery as cap water, and MFT consolidation

The height of the mud line was used to measure solids consolidation and calculate porewater recovery. The volumes of cap water (V_cw_) and MFT (solids plus porewater plus gas below the mud line; V_MFT_) were determined, respectively, by measuring the height of the cap water level above the mud line and height of the mud line in each column. The measured heights were converted to volumes using the following equation:

$$\text{V}=\pi r^{2}h_{\text{mud line}}$$

where, V = volume of cap water or MFT,

r = inner radius of the column,

h_mud line_ = height of the cap water level or mud line.

Then, water recovery (WR) defined as the cap water volume (above the mud line) was calculated as a percentage of the initial volume of porewater in MFT:

$$\text{WR}=({\text{V}}_{\text{cw}}/{\text{V}}_{\text{in}})\times 100$$

where, WR = water recovery (%),

V_cw_ = measured volume of cap water,

V_in_ = initial volume of porewater MFT.

Consolidation of MFT is defined as the MFT volume (below the mud line) calculated as a percentage of the initial volume of MFT

$$\text{Consolidation }(\%)=[({\text{V}}_{\text{in}}-{\text{V}}_{\text{MFT}})/{\text{V}}_{\text{in}}]\times 100$$

where, V_MFT_ = measured volume of MFT and V_in_ = initial volume of MFT.

#### Estimation of MFT consolidation in 50-L columns by deducting volume of entrapped gas bubbles

Estimated consolidation of MFT in unamended and amended columns was calculated using the following equation:

$$\text{Consolidation }(\%)=[({\text{V}}_{\text{in}}-{\text{V}}_{\text{MFT-d}})/{\text{V}}_{\text{in}}]\times 100,$$

where, V_in_ is the initial volume of MFT (45 L), and V_MFT−d_ is volume of MFT without gas at different densities that are calculated as:

$${\text{V}}_{\text{MFT-d}}=({\text{m}}_{\text{MFT-In}}-{\text{m}}_{\text{wr}})/\text{D}$$

where, m_MFT−In_ is the initial mass of MFT (54.9 kg); m_wr_ is the mass of released water. D is the density of the MFT on different days, determined from the mean weight of five replicate 10-mL subsamples of MFT.

#### Volume and composition of gas emitted by ebullition

The volume of gas emitted from 50-L columns was measured using a NaCl-citric acid brine gas trap (Boone, 1982). The composition of produced gas for CH_4_ and CO_2_ was determined using a Varian CP-2003 portable Micro-GC (gas chromatograph) equipped with a GMT-2HP moisture trap and thermal conductivity detector (TCD). The minimum detection limit of this GC was 50 ppm.

#### pH and redox potential (E_h_)

The pH was determined *in situ* at intervals during incubation by inserting a pH meter (Hach H170multi) fitted with an ISFET pH stainless steel micro probe (PHW17-SS) through side ports (Figure 1). For E_h_ measurements, MFT samples were collected from ports 2 and 3 under a continuous flow of N_2_ and immediately subjected to E_h_ measurement using a TX100 pH/mV-meter fitted with redox-sensitive electrode (Sensorex 651CD-ORP).

#### Soluble cations and anions

MFT samples removed from the columns were centrifuged (3075 *g* for 1 h) in a Sorvall RC 5B Superspeed centrifuge to collect the porewater. The solids fraction was analyzed as described below. For the soluble cations calcium (Ca^2+^), magnesium (Mg^2+^), sodium (Na^+^) and potassium (K^+^), porewater was filtered and diluted with 1% HNO_3_ (trace metal grade) and analyzed using Inductively Coupled Plasma Mass Spectrometry (ICP-MS; Perkin Elmer SCIEX ELAN 9000) with appropriate internal and external standards. Chloride (Cl^−^), nitrate (NO^−^_3_) and sulfate (SO^2−^_4_) soluble anion concentrations were determined using an ion chromatograph (Dionex DX 600) equipped with a 4 mm analytical column (AS9-HC). Dissolved bicarbonates (HCO^−^_3_) were determined using the methyl orange indicator method (US EPA, 1974) and Smartchem Discrete Wet Chemistry Analyzer 200 (Westco Scientific) at 550 nm. The PO^3−^_4_ concentration was determined using the colorimetric Molybdenum Blue method (US EPA, 1993) using the SmartChem at λ 880 nm.

#### Moisture correction factor

Data from chemical analyses of the solid phase (carbonates and exchangeable cations) were corrected for moisture and expressed on a solid dry weight basis. Moisture content in the samples was determined by drying in an oven at 105°C to constant weight (ISO 11465, 1993). The moisture content was obtained in weight percent and the corresponding correction factor was developed using the following equation:

Moisture correction factor (MCF)=(100+moisture %)/100

#### Exchangeable cations

Five grams of MFT were collected in 50-mL polypropylene centrifuge tubes (Corning Inc.) and 20 mL of 0.1M BaCl_2_/0.1M NH_4_Cl solution was added to displace the exchangeable cations (Sparks, 1996; Carter and Gregorich, 2008; Rayment and Lyons, 2012). The samples were shaken for 2 h and centrifuged (3075*g* for 1 h) in a Sorvall RC 5B Superspeed centrifuge. The supernatant was diluted with 1% HNO_3_ (trace metal grade) and analyzed using ICP-MS. In a few cases where the samples were not analyzed immediately after centrifugation, the supernatant was preserved by acidification to pH 1–2 using HNO_3_. The concentrations of exchangeable cations were calculated by subtracting concentrations of soluble cations (So et al., 2006). The results were expressed on dry weight basis.

#### Ionic strength (i) and diffuse double layer (DDL)

Ionic strength of the MFT porewater was calculated using the following formula (Essington, 2004):

$$\text{I}=1/2\sum_{i}\left({\text{M}}_{i}{\text{Z}}_{i}^{2}\right)$$

where, M*_i_* is the molar concentration of charged species (*i*) with charge Z*_i_* in the porewater and the sum includes all charged species measured in the porewater.

The thickness of the DDL was calculated using the following formula (Essington, 2004):

$${\text{k}}^{-1}=(3.042\times 10^{-8})/\text{Z}I^{0.5}$$

where, k^−1^ is the thickness (cm) of the DDL, Z is the average mean charge of the counterions (exchangeable cations), and *I* is the ionic strength of the porewater. Because MFT is a polyionic system having ions of different charges, the average mean charge of the exchangeable cations was calculated using the following equation:

$${\text{Z}}_{\text{ave}}=\sum {\text{C}}_{i}{\text{Z}}_{i}/\sum {\text{C}}_{i}$$

where, C*_i_*is the concentration of ions on the exchangeable surface and Z*_i_* is the charge of the species (*i*).

#### Carbonate minerals

The total carbonates in the MFT solid phase were determined by acid digestion (Pansu and Gautheyrou, 2006). The MFT (~10 mL) was centrifuged to separate the solid phase which was washed twice with 30 mL methanol to remove any soluble HCO^−^_3_. The MFT solid phase (~2 g) was placed in a serum bottle capped with a butyl rubber stopper and the headspace in the bottle was flushed with N_2_ at atmospheric pressure. The carbonates were dissolved by adding 20 mL of 1 M HCl to the bottles using a syringe. The bottle was gently shaken for 2 h and the amount of CO_2_ released was measured using GC-TCD (HP 5890). After CO_2_ measurement, the contents of the bottle were analyzed for Ca and Mg using an Atomic Absorption Spectrometer (AAS; SpectrAA 880). These concentrations were used to calculate CaCO_3_ and CaMg(CO_3_)_2_ in the MFT.

## Results

**Figure 2:**
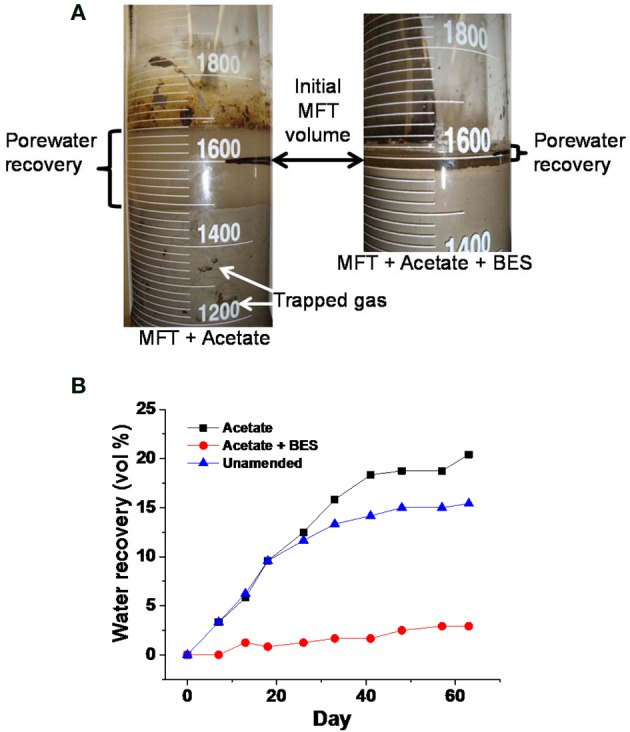
**Effect of adding 10 mM sodium 2-bromoethanesulfonate (BES), a specific inhibitor of methanogens, on dewatering of 1.6 L MFT in 2-L columns incubated undisturbed for >60 d at ambient temperature (~22°C)**. **(A)** Photographs of porewater recovery and total volume of MFT solids and porewater, including trapped biogenic gases (white arrows) in parallel columns containing MFT amended with acetate (140 mg L^−1^ MFT) or with acetate plus BES (few, tiny bubbles; not visible), or unamended (no bubbles; not shown). **(B)** Quantitation of porewater recovery from MFT amended with acetate, or with acetate plus BES, or unamended, illustrating the magnitude of the microbial effect (Figure is constructed using the data retrieved from Bressler et al. 2010).

We investigated contributions of microbial activity (methanogenesis) to consolidation in a series of experiments conducted at different scales from 2-L to 50-L column studies. The results reveal that methanogenesis is important for rapid consolidation of MFT, an effect that is superimposed on tailings self-weight consolidation. Acetate, a well-known substrate for methanogenesis, resulted in greater porewater recovery and solids consolidation when added to MFT as compared to unamended MFT (Figure 2; constructed using data retrieved from Bressler et al., 2010). The same phenomenon has also been observed in our previous experiments (Arkell et al., in review; Young et al., in preparation). The increase in total volume in the acetate-amended MFT shown in Figure 2A, left panel is due to biogas entrained in the MFT solids. However, specifically inhibiting methanogenesis by adding sodium 2-bromoethanesulfonate (BES) resulted in minimal consolidation, presumably equivalent to self-weight settling, minimal porewater recovery and no visible pockets of entrained gas (Figure 2). Thus, it appears that active methanogenesis is a prerequisite for biodensification of MFT incubated under these conditions.

**Figure 3:**
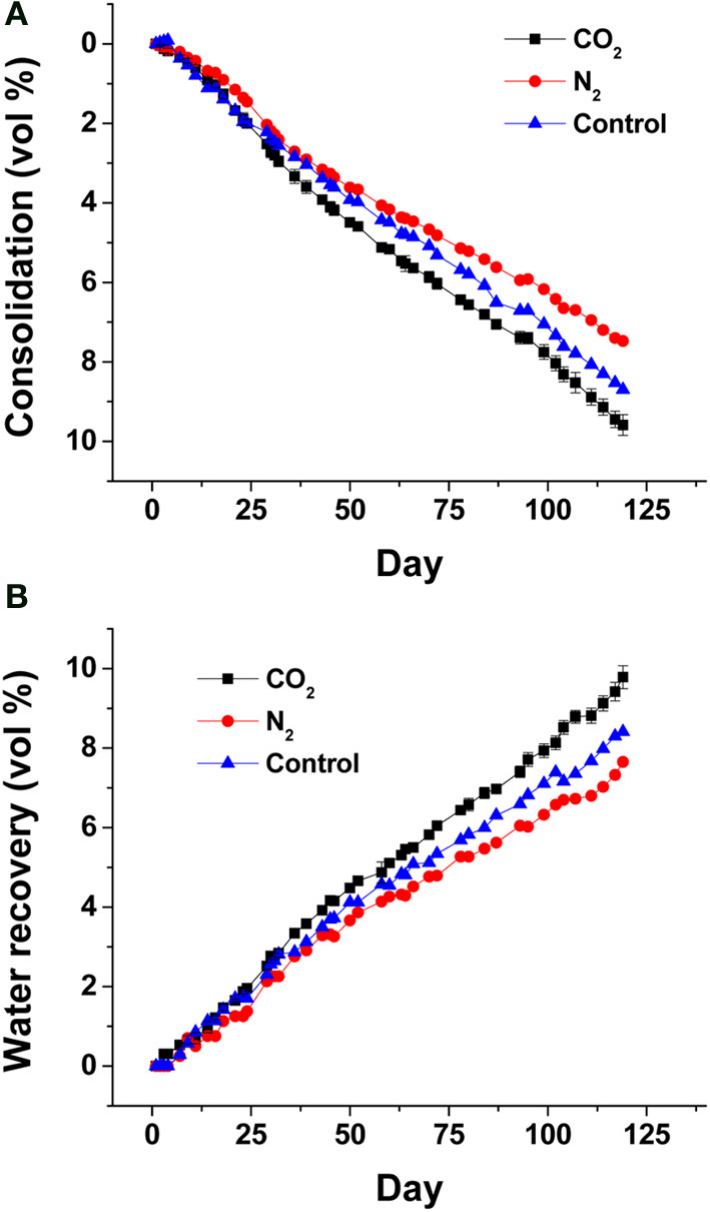
**Effect of abiotic gas purging on MFT consolidation (A) and porewater recovery (B)**. Four replicate 2-L columns containing 1.7 L unamended MFT were purged with CO_2_ (*n* = 2; error bars, where visible, show the standard error) or N_2_ (*n* = 1) or no gas purging (*n* = 1) prior to dispensing MFT into columns. Consolidation was calculated from the volume of solids below the mud line as a fraction of the total volume of MFT plus water plus any trapped gas. Porewater recovery was calculated using the recovered porewater volume above the mud line (i.e., cap water volume) as a fraction of the initial total volume of porewater in MFT.

To further demonstrate the contribution of biogenic vs. abiotic gases to porewater recovery and consolidation, small-scale (2-L column) experiments were performed in which unamended MFT was sparged with CO_2_ to simulate biogenic CO_2_ production, or with N_2_ gas to simulate the effect of insoluble gas ebullition (by creating transient physical channels for expression of porewater). Only marginal differences in porewater recovery and tailings consolidation were observed compared to non-sparged MFT (Figure 3). The small proportion and slow rates of consolidation and porewater recovery (compared to acetate-amended MFT; Figure 2) presumably are due to methanogenic activity supported by endogenous organic substrates in MFT.

### Biogenic gas production and decreased pH affect porewater recovery from MFT in 50-L columns

**Figure 4:**
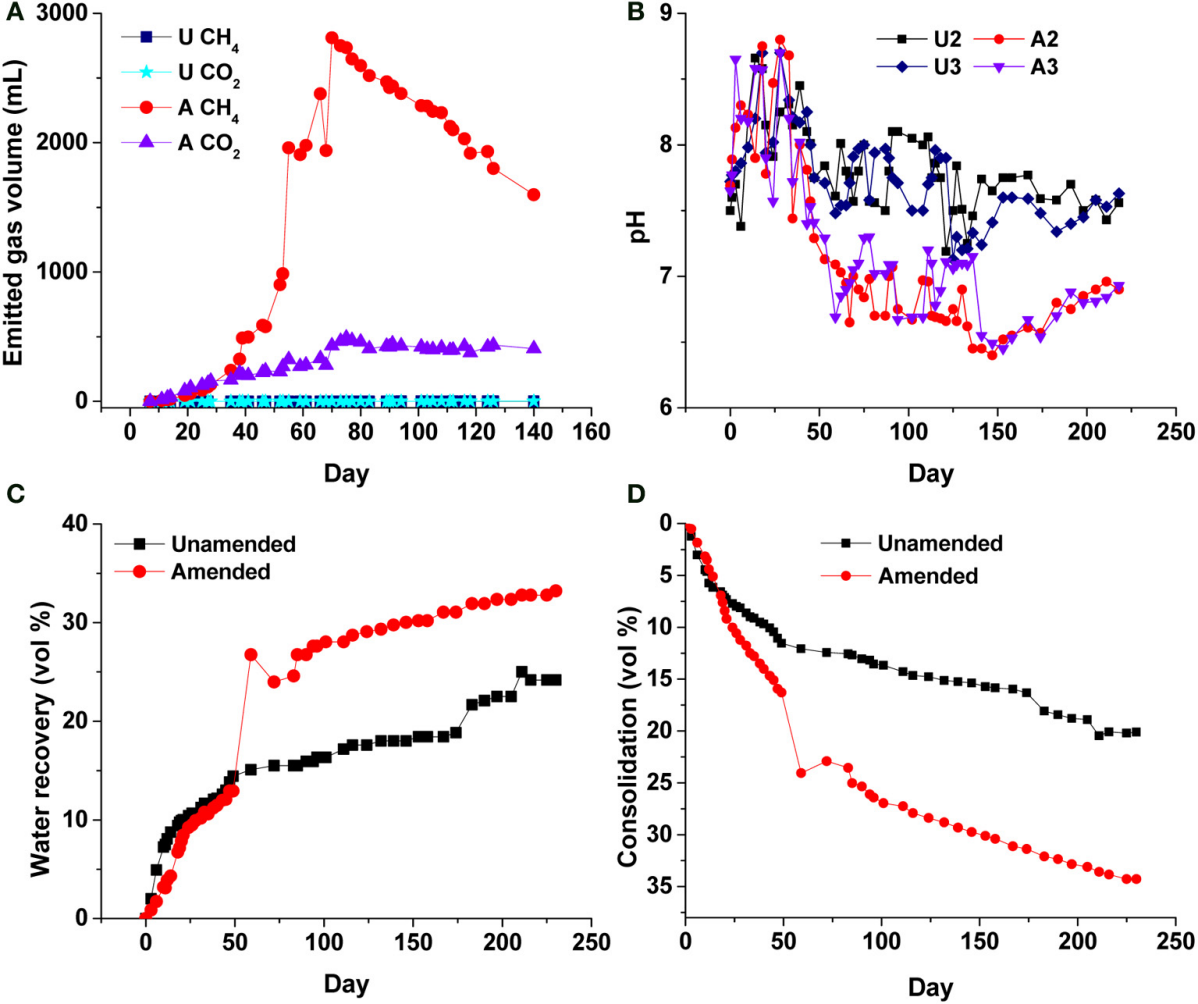
**Physico-chemical changes to unamended (U) and amended (A) MFT in 50-L columns**. **(A)** Cumulative emitted methane (CH_4_) and carbon dioxide (CO_2_) in headspace (not accounting for gases trapped or dissolved in MFT); **(B)** bulk pH of MFT, measured at ports 2 and 3; **(C)** Proportion of initial porewater recovered above the mud line as cap water (i.e., expressed porewater). The sudden increase in cap water in the amended column corresponded to a large ebullition event at ~50 days recorded in **A**; **(D)** Consolidation of solids, corrected for trapped gas volumes by subtracting the estimated volume of trapped gas from the measured total MFT volume. Initial and final densities of unamended (*D* = 1.22 and 1.30 g mL^−1^, respectively) and amended MFT (*D* = 1.22 and 1.49 g mL^−1^, respectively) were used to calculate consolidation; see Methods for details.

In previous small-volume experiments we used complex or simple fermentable organic amendments to accelerate dewatering and consolidation of MFT (Bressler et al., 2010; Arkell et al., in review; Young et al., in preparation), and determined that a low concentration of hydrolyzed canola meal (400 mg C L^−1^ MFT) was sufficient to promote tailings biodensification under methanogenic conditions. In the current experiment, it is apparent that methanogenic conditions were established in the 50-L columns, as anaerobic metabolism of the soluble canola hydrolysate resulted in emission of ~2.8 L CH_4_ and ~0.5 L CO_2_ (CH_4_:CO_2_ ratio of 5.6:1) to the headspace by 75 d, with minor gas production from unamended MFT (Figure 4A). Additional biogenic gas was trapped within amended MFT (Figure S1 Panel B), contributing to the total apparent volume of MFT. Stoichiometric calculation of theoretical gas production from canola, based on the Symons and Buswell equation (Roberts, 2002), predicted a CH_4_:CO_2_ ratio of ~3:1, but substantially less CO_2_ than predicted was measured in the headspace (Figure 4A). The deficit in emitted CO_2_ is explained by dissolution of biogenic CO_2_ in porewater, which decreased the pH of amended MFT from 7.7 to 6.4 by 147 d, whereas the pH of unamended MFT was essentially unchanged in >200 d (Figure 4B). We initially observed an increase in pH in the amended MFT during the first 35 d, after which it significantly decreased. During active gas biogenesis in the first 2 months, 12% more porewater was released from amended MFT than unamended (Figure 4C), most of which coincided with pH reduction, a major ebullition event at 50 d, and rapid consolidation of amended MFT. Ebullition of biogenic CH_4_ created transient channels for escape of porewater from MFT, observed particularly near the mud line (MFT–cap water interface; Figure S1). Other experiments (unpublished) confirmed that transient water- and gas-filled channels can form in MFT to allow passage of porewater to the surface during this phase. Gas bubbles remaining in the amended MFT did not affect subsequent consolidation behavior (Figure S2), but did contribute to the apparent total solids volume; complete degassing theoretically would have yielded ~15 vol% more consolidation in the amended column (Figure 4D).

### pH affects dissolution of carbonate minerals and porewater chemistry

**Figure 5:**
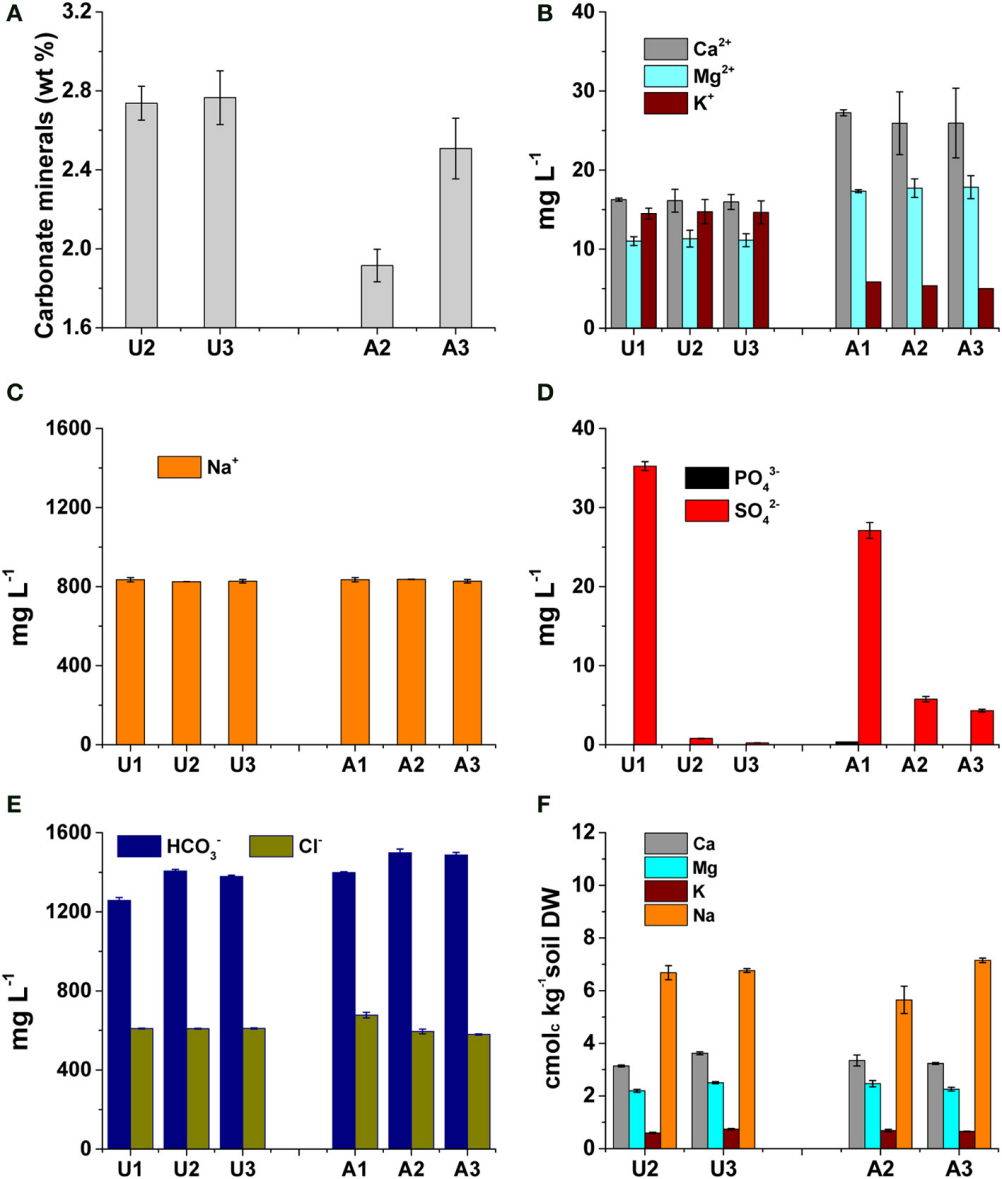
**Concentrations of carbonate minerals and major cations and anions in unamended (U) and amended (A) 50-L columns after 213 d incubation**. Labels 1, 2, and 3 refer to samples collected from ports on the columns (see Figure 1); Port 1 accessed cap water only by 213 d; ports 2 and 3 were below the mud line and accessed MFT. Bars represent the mean from analyses of duplicate or triplicate samples taken from each port and error bars, where visible, represent 1 standard deviation. **(A)** Carbonate mineral content of MFT samples (*n* = 3). **(B,C)** Concentrations of major soluble cations in cap water and interstitial porewater (*n* = 2). **(D,E)** Concentrations of major soluble anions in cap water and interstitial porewater (*n* = 2). **(F)** Concentrations of exchangeable cations in MFT (*n* = 2).

The decreased pH in amended MFT due to dissolution of biogenic CO_2_ increased dissolution of carbonate minerals in MFT (Figure 5A): at 213 d, the carbonate mineral content in amended MFT (1.7 wt%) was significantly lower than unamended MFT (2.7 wt%). Carbonate dissolution increased soluble cation concentrations in porewater and cap water: Ca^2+^ and Mg^2+^ concentrations were greater in amended MFT (~32 and 19 mg L^−1^, respectively) than unamended MFT (~17 and 10 mg L^−1^, respectively; Figure 5B). In contrast, soluble K^+^ decreased in amended MFT (Figure 5B) whereas the soluble Na^+^ concentration remained unaffected in amended MFT porewater (Figure 5C). A very small concentration of soluble phosphate (PO^3−^_4_) was detected only in amended MFT porewater (0.36 mg L^−1^) with low concentrations of sulfate (SO^2−^_4_, <5 mg L^−1^) (Figure 5D). Amended MFT had slightly higher porewater concentrations of soluble HCO^−^_3_ (~1500 mg L^−1^) than unamended MFT (~1380 mg L^−1^) but Cl^−^ concentrations were comparable in both amended and unamended MFTs (Figure 5E).

### Exchangeable cations, ionic strength and diffuse double layer (DDL)

**Figure 6:**
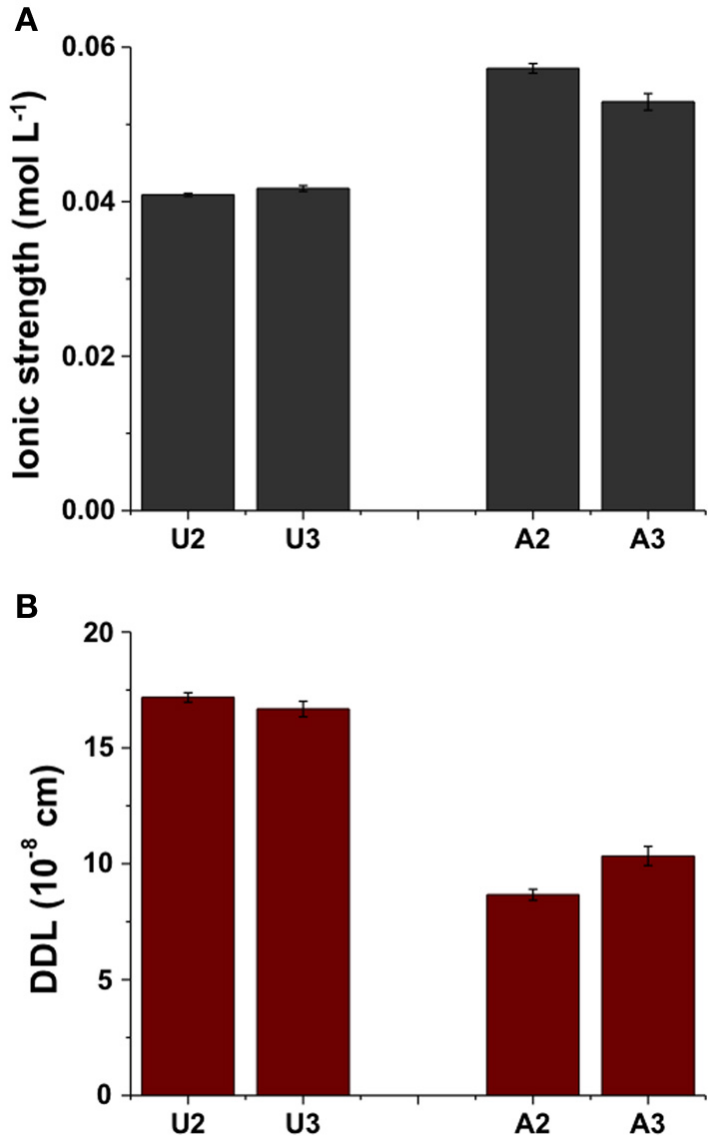
**(A)** Calculated ionic strength (*I*) of the porewater and **(B)** thickness of diffuse double layer (DDL) of clay particles in unamended (U) and amended (A) MFT in the 50-L columns at ports 2 and 3 (Figure 1). Ionic strength was calculated using the data presented in Figure 5, the DDL thickness was calculated using the ionic strength, and charges of exchangeable cations were calculated from data shown in Figure 5F. Bars represent the mean values from analyses of duplicate samples taken from each port and error bars, where visible, represent 1 standard deviation.

Although dissolution of carbonates increased Ca^2+^ and Mg^2+^ concentrations in porewater of amended MFT, no significant change in the composition of exchangeable cations on clay surfaces was observed in MFT (Figure 5F). However, increased concentrations of soluble cations (Ca^2+^ and Mg^2+^) and anions (HCO^−^_3_) increased the ionic strength (*I*) of the porewater (Figure 6A). The greater *I* (~0.055 mol L^−1^) was calculated for the porewater from amended MFT vs. unamended MFT (~0.04 mol L^−1^). The *I* of a solution has a profound effect on the DDL of clay particles. Increased *I* in the porewater of amended MFT decreased the DDL thickness (~10 × 10 ^−8^ cm) of clay particles in amended MFT as compared to the DDL thickness (~18 × 10^−8^ cm) calculated for clay particles in unamended MFT (Figure 6B).

## Discussion

Dewatering of tailings is a major challenge faced by the surface-mining oil sands industry to comply with government directives for managing the ever-growing inventory of oil sands tailings (http://www.aer.ca/rules-and-regulations/directives/directive-074). Any process that increases the settling of clay particles in oil sands tailings ponds will enhance consolidation of tailings to reduce the inventory volume and enable reclamation, and will increase recovery of porewater from MFT for re-use and reduction of fresh water demand; even marginal increases in consolidation translate into enormous volumes, given the scale of existing tailings ponds (>920 million m^3^). Thus, microbially-mediated consolidation and dewatering (biodensification) can mitigate this problem, whether supported by endogenous substrates *in situ* (Fedorak et al., 2003) or by amendment with organic substrates in an engineered process *ex situ*. In this paper, we have focused on how microbial activities alter porewater chemistry to enhance tailings consolidation and dewatering; in the companion paper (Siddique et al., 2014), we consider the effect on tailings solids.

**Figure 7:**
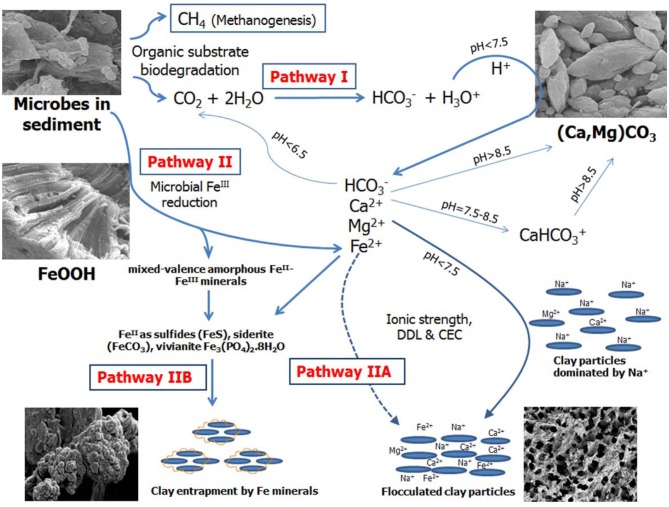
**Proposed model for microbially-mediated geochemical pathways of clay consolidation**. This study focuses on Pathway I; the companion paper (Siddique et al., 2014) focuses on Pathway II. Dissolution of biogenic CO_2_ in tailings decreases porewater pH by producing hydronium ion (H_3_O^+^). Increased H_3_O^+^ dissolves carbonate minerals such as CaMg(CO_3_)_2_ and releases calcium (Ca^2+^) and magnesium (Mg^2+^). Dissolution of CO_2_ and/or carbonate minerals increases bicarbonate (HCO^−^_3_) ions in porewater. Liberated Ca^2+^ and Mg^2+^ plus HCO^−^_3_ increase ionic strength (*I*) of porewater, thus reducing the diffuse double layer (DDL) of clay particles and facilitating their consolidation. Ca^2+^ and Mg^2+^ can replace some dominant exchangeable Na from clay exchanger surfaces via the cation exchange process, reducing DDL of clay particles. Competing reactions are shown by solid arrows while dashed arrows indicate pathways not considered significant in our study.

The mechanism of microbially-mediated consolidation (biodensification) of oil sands tailings is multi-faceted, embracing different biogeochemical processes occurring simultaneously in the MFT (Figure 7). Stimulating the activity of indigenous anaerobes by amending MFT with labile organic carbon (hydrolyzed canola meal in this study, acetate in previous studies; Fedorak et al., 2003; Arkell et al., in review) enhanced biogenic CH_4_ and CO_2_ production. Gas production resulted in ebullition of bubbles dominated by CH_4_ (due to poor solubility of CH_4_ in water), creating transient channels for escape of pressurized porewater, particularly in MFT near the mud line. Methanogenesis in MFT might be responsible for the observed initial pH increase as acetate and CO_2_ were consumed (Fotidis et al., 2013) but the dissolution of entrapped CO_2_ reduced porewater pH, thereby dissolving carbonate minerals and releasing divalent cations (Pathway I in Figure 7). Our results are very similar to the findings of Wersin et al. (2011) who studied biogeochemical processes in a clay formation *in situ*. They observed carbonate dissolution, high pCO_2_ and alkalinity (HCO^−^_3_ and CO^2−^_3_) and a decrease in pH from 7.7 to 6.8 during anaerobic degradation of an organic substrate under sulfate-reducing and methanogenic conditions. Similar results were also reported by Schlegel et al. (2011) studying fluid geochemistry and microbiology of multiple organic-rich reservoirs in the Illinois Basin, USA. Our results are also supported by other studies where the influence of benthic bacterial activity on carbonate mineral dissolution in marine sediments has been described (Moulin et al., 1985) and dissolution of marine carbonate minerals by acidification due to increased atmospheric CO_2_ has been comprehensively reviewed (Morse et al., 2007). Solubility of CaCO_3_ increases 20-fold between pH 8 and pH 6 (Chou et al., 1989) and siderite can be dissolved in anaerobic aqueous environments at pH~7 (Jensen et al., 2002).

Dissolution of MFT carbonate minerals (presumably calcite/dolomite) increased Ca^2+^ and Mg^2+^ concentrations in amended porewater, whereas K^+^ decreased in amended MFT, which might be attributed to reducing conditions prevailing in amended MFT (Siddique et al., 2014) facilitating K^+^ fixation in reduced 2:1 phyllosilicates (Eslinger et al., 1979; Shen and Stucki, 1994). Phyllosilicate analysis of MFT showed that fine clay (0.08–0.2 μm) comprised ~45% kaolinite-smectite (94% kaolinite) and 55% illite-smectite (93% illite) group minerals whereas clay (0.2–2 μm) consisted of 62% kaolinite-smectite (100% kaolinite) and 36% illite-smectite (96% illite) groups (Siddique et al., 2014). Among anions, a relatively higher concentration of HCO^−^_3_ in the porewater of amended MFT was observed, presumably due to dissolution of biogenic CO_2_ in porewater and/or dissolution of carbonate minerals (Morse et al., 2007). The change in porewater chemistry of amended MFT due to microbial metabolism did not alter the cationic composition on clay exchanger surfaces despite significant increases in divalent cation concentrations (Ca^2+^ and Mg^2+^) in the porewater. However, the divalent cation concentrations were dwarfed by the Na^+^ concentration, which dominated the porewater composition. Another possible reason for the apparent lack of cation exchange could be technical, since no method has been optimized yet to determine the exchangeable cations in oil sands tailings. Although we cannot exclude cation exchange processes based on our results, we do not consider cation exchange to be a significant or essential process in MFT consolidation (Pathway I, Figure 7, broken lines).

The phenomenon of clay dispersion in aqueous systems is understood using the concept of diffuse double layer (DDL), the thickness of which is governed by the charge potential on the clay surface (one electric layer, generally with net negative charge on clay surface) and valence of the counterions in solution (second electric layer, positive charge contributed by cations) neutralizing the surface charge potential of clay. Decreasing thickness of DDL increases clay flocculation. In our study, the greater ionic strength (*I*) of porewater from amended MFT, attributed to increased concentrations of soluble ions (Ca^2+^, Mg^2+^, and HCO^−^_3_), would decrease the thickness of the DDL of clay particles (Figure 6) and enhance consolidation of tailings. Electrostatic repulsion between the clay particles is reduced at higher *I* due to ion shielding of the clay surface charges, decreasing thickness of the DDL. Greater consolidation of amended MFT might also be attributed to dominance of kaolinite phyllosilicates in the MFT, in addition to greater *I*. The charges on kaolinite are largely pH-dependent, and the observed lower pH of MFT would decrease the net negative charges on the clay surfaces. Nasser and James (2006) examined the settling behavior of kaolinite in aqueous media in response to *I* and found that, at higher *I*, particles settled in flocculated form. Similar results were reported by Mietta et al. (2009) for flocculation of a kaolinite suspension as function of pH and salt concentration, and they found that clay floc size was 3-fold greater at pH 4 than at pH 7. Our small-scale, short-term (24-h) abiotic experiment conducted with unamended MFT confirmed that adding different salts or decreasing bulk pH by adding acid markedly increased consolidation and porewater recovery from tailings (Figure S3). Greater consolidation occurred in MFT amended with FeCl_2_ compared to untreated MFT, followed by the effects of CaCl_2_ and then NaCl; maximum consolidation was achieved using 6N HCl (pH 1). These results support our proposed biogeochemical model (Pathway I, Figure 7), in that: (1) increased *I* of the MFT decreased DDL of clay particles; (2) divalent cations can exchange monovalent cations from exchanger surfaces (although not observed in current study, this was a significant effect in an acetate-amended 2-L MFT column study; Arkell et al., in review), thereby decreasing DDL; and (3) lower MFT pH dissolved carbonate minerals and changed pH-dependent charges on clays, leading to consolidation of MFT.

The results of this study are important for the management of oil sands tailings. Microbially-enhanced tailings consolidation apparently is occurring *in situ* (Fedorak et al., 2003), likely supported by endogenous substrates such as light hydrocarbons used in bitumen extraction, where concomitant recovery of porewater can decrease on-site demand for freshwater for oil sand ore processing. Biodensification may also facilitate dry landscape reclamation of MFT by complementing or possibly foregoing current chemical and physical management practices like chemical flocculant addition and energy-intensive centrifugation processes. In addition to oil sands tailings, it would be prudent to consider the roles of indigenous microbes in the consolidation of other organic-rich soft sediments, such as those in contaminated harbors and river deltas, and to incorporate biological factors into consolidation models.

### Conflict of interest statement

The authors declare that the research was conducted in the absence of any commercial or financial relationships that could be construed as a potential conflict of interest.

## References

[B1] Alberta Environment and Sustainable Resource Development. (2013). Oil sands landcover status 2011. Edmonton, AB: AERSD 1995.

[B2] Boone D. R. (1982). Terminal reactions in the anaerobic digestion of animal waste. Appl. Environ. Microbiol. 43, 57–64 16345928 10.1128/aem.43.1.57-64.1982 PMC241780

[B3] Bressler D. Cardenas M. Fedorak P. M. Guigard S. Gupta R. Siddique T. (2010). Microorganisms in oil sand tailings ponds influence the properties and behaviour of mature fine tailings, in Proceedings of 2nd International Oil Sands Tailings Conference (Edmonton, AB), 44.

[B4] Brown D. Ramos-Padrón E. Gieg L. Voordouw G. (2013). Effect of calcium ions and anaerobic microbial activity on sedimentation of oil sands tailings. Int. Biodeterior. Biodegradation 81, 9–16 10.1016/j.ibiod.2012.07.006

[B5] Carter M. R. Gregorich E. G. (2008). Soil Sampling and Methods of Analysis. Can. Soc. Soil Sci. Oxford: CRC Press

[B6] Chou L. Garrels R. M. Wollast R. (1989). Comparative study of the kinetics and mechanisms of dissolution of carbonate minerals. Chem. Geol. 78, 269–282 10.1016/0009-2541(89)90063-6

[B7] Dean E. W. Stark D. D. (1920). A convenient method for the determination of water in petroleum and other organic emulsions. Ind. Eng. Chem. 12, 486–490 10.1021/ie50125a025

[B8] Eslinger E. Highsmith P. Albers D. De Mayo B. (1979). Role of iron reduction in the conversion of smectite to illite in bentonites in the disturbed belt, Montana. Clays Clay Miner. 27, 327–338 10.1346/CCMN.1979.0270503

[B9] Essington M. E. (2004). Soil and Water Chemistry: An Integrative Approach. Boca Raton, FL: CRC Press LLC

[B10] Fedorak P. M. Coy D. L. Dudas M. J. Simpson M. J. Renneberg A. J. MacKinnon M. D. (2003). Microbially-mediated fugitive gas production from oil sands tailings and increased tailings densification rates. J. Environ. Eng. Sci. 2, 199–211 10.1139/s03-022

[B11] Fotidis I. A. Karakashev D. Kotsopoulos T. A. Martzopoulos G. G. Angelidaki I. (2013). Effect of ammonium and acetate on methanogenic pathway and methanogenic community composition. FEMS Microbiol. Ecol. 83, 38–48 10.1111/j.1574-6941.2012.01456.x 22809020

[B12] Himmelheber D. W. Pennell K. D. Hughes J. B. (2011). Evaluation of a laboratory-scale bioreactive *in situ* sediment cap for the treatment of organic contaminants. Water Res. 45, 5365–5374 10.1016/j.watres.2011.06.022 21872291 PMC3183260

[B13] ISO 11465. (1993). Soil Quality – Determination of Dry Matter and Water Content on a Mass Basis – Gravimetric Method. Geneva: Int. Org. Standard

[B14] Jeeravipoolvarn S. Scott J. D. Chalaturnyk R. J. (2009). 10 m standpipe tests on oil sands tailings: long term experimental results and prediction. Can. Geotech. J. 46, 875–888 10.1139/T09-033

[B15] Jensen D. L. Boddum J. K. Tjell J. C. Christensen T. H. (2002). The solubility of rhodochrosite (MnCO_3_) and siderite (FeCO_3_) in anaerobic aquatic environments. Appl. Geochem. 17, 503–511 10.1016/S0883-2927(01)00118-4

[B16] Mietta F. Chassagne C. Winterwerp J. C. (2009). Shear-induced flocculation of a suspension of kaolinite as function of pH and salt concentration. J. Colloid Interface Sci. 336, 134–141 10.1016/j.jcis.2009.03.044 19423126

[B17] Morse J. W. Arvidson R. S. Lüttge A. (2007). Calcium carbonate formation and dissolution. Chem. Rev. 107, 342–381 10.1021/cr050358j 17261071

[B18] Moulin E. Jordens A. Wollast R. (1985). Influence of the aerobic bacterial respiration on the early dissolution of carbonates in coastal sediments, in Progress in Belgian Oceanographic Research, eds Van Grieken R. Wollast R. (Brusells: University of Antwerpen), 196–208

[B19] Nasser M. S. James A. E. (2006). The effect of polyacrylamide charge density and molecular weight on the flocculation and sedimentation behaviour of kaolinite suspensions. Sep. Purif. Technol. 52, 241–252 10.1016/j.seppur.2006.04.005

[B20] Pansu M. Gautheyrou J. (2006). Handbook of Soil Analysis. Mineralogical, Organic and Inorganic Methods. Berlin; Heidelberg: Springer-Verlag 10.1007/978-3-540-31211-6

[B21] Penner T. J. Foght J. M. (2010). Mature fine tailings from oil sands processing harbour diverse methanogenic communities. Can. J. Microbiol. 56, 459–470 10.1139/W10-029 20657616

[B22] Rayment G. E. Lyons D. J. (2012). New, comprehensive soil chemical methods book for Australasia. Commun. Soil Sci. Plant Anal. 43, 412–418 10.1080/00103624.2012.641802

[B23] Roberts D. J. (2002). Methods for assessing anaerobic biodegradation potential, in Manual of Environmental Microbiology, eds Hurst C. J. Crawford R. L. Knudson G. R. McInerney M. J. Stetzenbach L. D. (Washington, DC: ASM Press), 1008–1017

[B24] Schlegel M. E. McIntosh J. C. Bates B. L. Kirk M. F. Martini A. M. (2011). Comparison of fluid geochemistry and microbiology of multiple organic-rich reservoirs in the Illinois Basin, USA: evidence for controls on methanogenesis and microbial transport. Geochim. Cosmochim. Acta 75, 1903–1919 10.1016/j.gca.2011.01.016

[B25] Schramm L. L. Stasiuk E. N. MacKinnon M. D. (2000). Surfactants in Athabasca oil sands slurry conditioning, flotation recovery, and tailings processes, in Surfactants, Fundamentals, and Applications in the Petroleum Industry, ed Schramm L. L. (Cambridge: Cambridge University Press), 365–430 10.1017/CBO9780511524844.011

[B26] Shen S. Stucki J. (1994). Effects of iron oxidation state on the fate and behavior of potassium in soils, in Soil Testing: Prospects for Improving Nutrient Recommendations, eds Havlin J. L. Jacobsen J. S. (Madison, WI: SSSA Special Publication 40), 173–185

[B27] Siddique T. Fedorak P. M. Foght J. M. (2006). Biodegradation of short-chain n-alkanes in oil sands tailings under methanogenic conditions. Environ. Sci. Technol. 40, 5459–5464 10.1021/es060993m 16999125

[B28] Siddique T. Fedorak P. M. Mackinnon M. D. Foght J. M. (2007). Metabolism of BTEX and naphtha compounds to methane in oil sands tailings. Environ. Sci. Technol. 41, 2350–2356 10.1021/es062852q 17438786

[B30a] Siddique T. Kuznetsov P. Kuznetsova A. Li C. Young R. Arocena J. M. (2014). Microbially-accelerated consolidation of oil sands tailings. Pathway II: solid phase biogeochemistry. Front. Microbiol. 5:107. 10.3389/fmicb.2014.00107 PMC3968759 24711806

[B29] Siddique T. Penner T. Klassen J. Nesbø C. Foght J. M. (2012). Microbial communities involved in methane production from hydrocarbons in oil sands tailings. Environ. Sci. Technol. 46, 9802–9810 10.1021/es302202c 22894132

[B30] Siddique T. Penner T. Semple K. Foght J. M. (2011). Anaerobic biodegradation of longer-chain n-alkanes coupled to methane production in oil sands tailings. Environ. Sci. Technol. 45, 5892–5899 10.1021/es200649t 21644510

[B31] So H. Menzies N. Bigwood R. Kopittke P. (2006). Examination into the accuracy of exchangeable cation measurement in saline soils. Commun. Soil Sci. Plant Anal. 37, 1819–1832 10.1080/00103620600762927

[B32] Sparks D. L. (1996). Methods of Soil Analysis: Part 3. Chemical Methods. Madison, WI: SSSA

[B33] US EPA. (1974). Method 310.2. Alkalinity (Colorimetric, Automated, Methyl Orange). Available online at: http://water.epa.gov/scitech/methods/cwa/methods_index.cfm

[B34] US EPA. (1993). Method 365.1 Revision 2.0. Determination of Phosphorous by Semi-Automated Colorimetry. Available online at: http://water.epa.gov/scitech/methods/cwa/methods_index.cfm

[B35] Voordouw G. (2013). Interaction of oil sands tailings particles with polymers and microbial cells: first steps toward reclamation to soil. Biopolymers 99, 257–262 10.1002/bip.22156 23348673

[B36] Wersin P. Leupin O. X. Mettler S. Gaucher E. C. Mäder U. De Cannière P. (2011). Biogeochemical processes in a clay formation *in situ* experiment: part A - overview, experimental design and water data of an experiment in the Opalinus Clay at the Mont Terri Underground Research Laboratory, Switzerland. Appl. Geochem. 26, 931–953 10.1016/j.apgeochem.2011.03.004

