# Supplementary material for: Microbially-accelerated consolidation of oil sands tailings. Pathway I: changes in porewater chemistry
Source: Front Microbiol. 2014 Mar 21;5:106. doi: 10.3389/fmicb.2014.00106 (PMC3968746; doi:10.3389/fmicb.2014.00106)
Supplement: Supplementary file 1 [file Presentation1.PDF]

APPENDIX

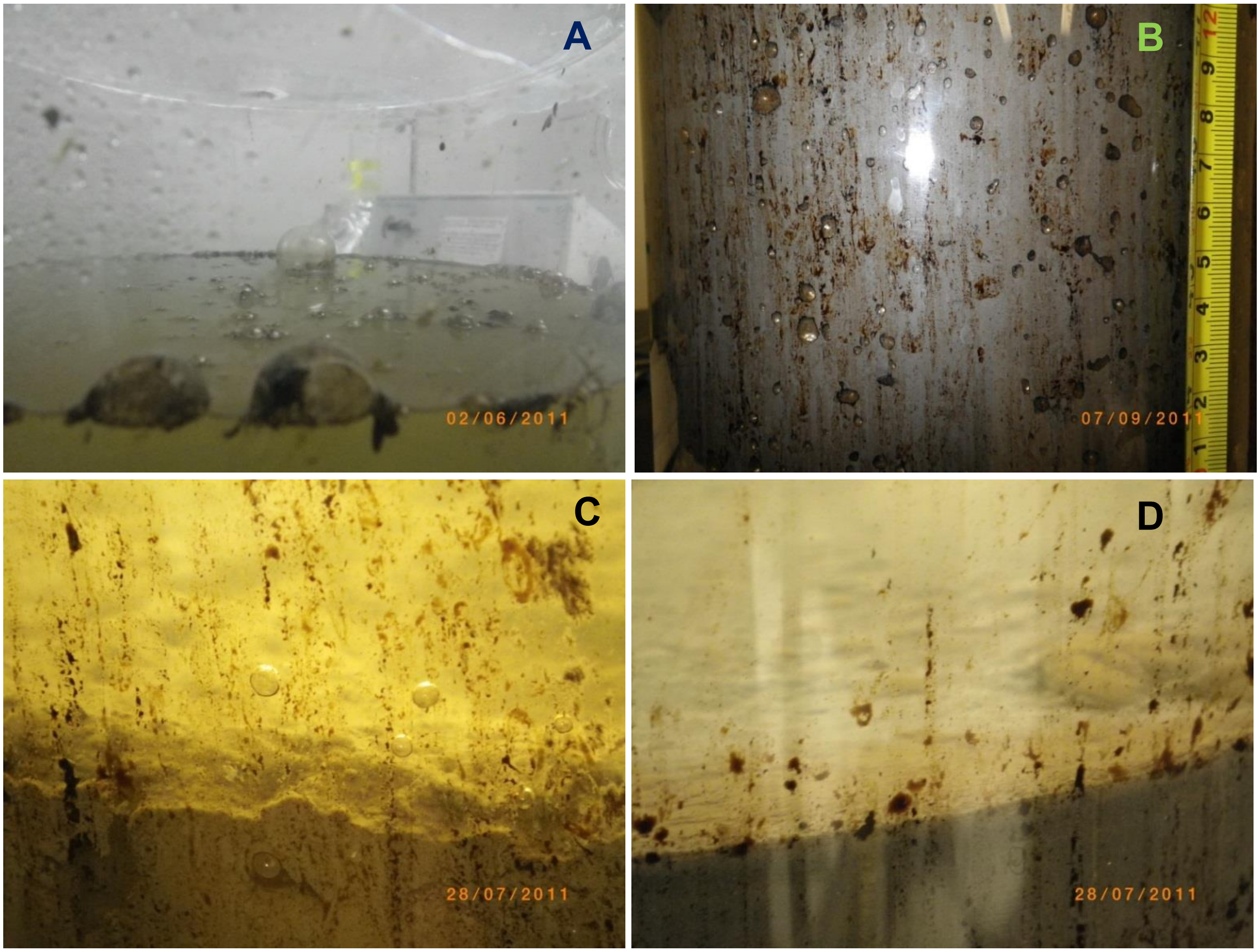

Fig. A1

Photographs of biogenic gas bubbling from and trapped in the 50-L columns during incubation. (**A**) Ebullition of gas from the surface of cap water in the amended column after 27 d incubation. (**B**) Gas bubbles trapped in amended MFT after 124 d. Dark streaks are residual bitumen (from MFT) adhering to the acrylic column. (**C**) Ebullition at the mud line (interface between cap water and MFT) in the amended column. (**D**) Absence of bubbles at mud line in the unamended column at 83 d.

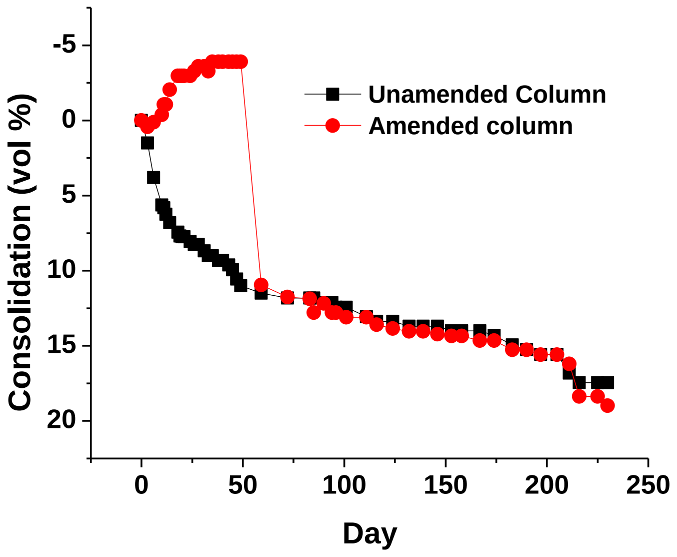

Fig. A2

Observed consolidation of MFT in the 50-L columns incubated for 213 days. The initial and final heights of the mud line (cap water-MFT interface) were used to calculate consolidation. The final calculated volume of MFT includes the trapped biogenic gases.

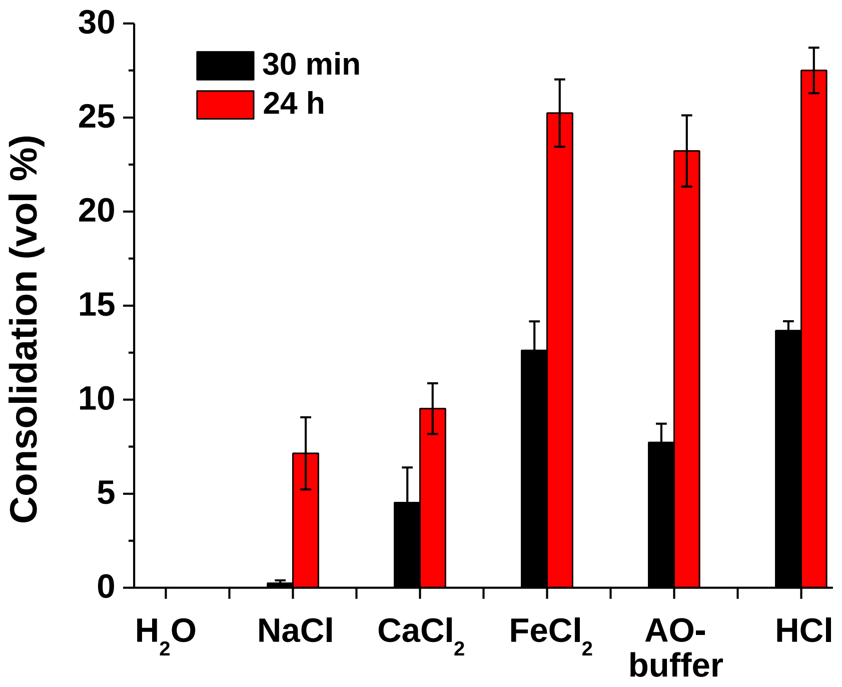

Fig. A3

Results from abiotic experiment measuring consolidation of MFT amended with different inorganic salt solutions, organic buffer, or inorganic acid, incubated for 30 min or 24 h. MFT was amended with an equal volume of 1N salt solutions (NaCl, $CaCl_2$ or $FeCl_2$), ammonium oxalate buffer (AO; pH 3) or 6N HCl (pH 1) or nano-pure water.
